# The Story of the Insane from Year to Year

**Published:** 1906-03-17

**Authors:** 


### THE STORY OF THE INSANE FROM YEAR TO YEAR.

Eighteenth Annual Series.

### HEREFORD COUNTY ASYLUM.

On January 1st this Asylum contained 513 patients, and on December 31st the number had risen to 527. There were 73 first admissions; 22 readmissions; 23 discharged recovered; 6 discharged relieved; 11 discharged not improved; and 41 deaths. The average number resident during the year was 512, and the total number under treatment was 599. Calculated on the admissions, the recoveries stand at 33.8 per cent., and calculated on the average number resident, the deaths were 8 per cent., both of these percentages being a little over the average for this Asylum, but under the general asylum average. Of the year's deaths 15 were caused by cerebral and spinal diseases; one by diphtheria, one by enteric fever, four by consumption, and three by other lung diseases. Table X shows that of the cases admitted 15 owed their insanity to such moral causes as domestic trouble and adverse circumstances; while among the physical causes hereditary tendency accounts for 23 cases, and intemperance in drink for six. Among the 35 men admitted during the year ten were general labourers; six were farm labourers; and one was a Wesleyan minister; and among the 60 women admitted 21 were housewives; 20 were of no occupation; and four were domestic servants. It would seem that the Hereford patients in the Asylum have increased by about eight per annum during the last ten years, and that the women's side of the Asylum has sufficient spare accommodation for several years to come; but on the men's side practically every bed is occupied, and we do not learn from this report what steps the visitors intend taking to prevent the overcrowding which is sure to follow. In this and in almost every asylum the strain is most felt in the infirm wards, and this will be the case until some change is made in the capitation grant received by the Guardians. When speaking of the admissions, Dr. Morrison says that "where the insanity is not the direct outcome of gross brain disease or atrophic senile changes, destructive to the delicate and unrenewable brain tissue, the age-period and duration of attack previous to institutional treatment form all important factors in the expectancy of a probable future recovery." Or, in other words, it is the speedy treatment of insanity that the public should keep in view; and its importance cannot be too often and too strongly stated. The chief structural improvement of the year was the provision of a new power-house. It has three Lancashire boilers and three dynamos. Dr. Morrison has two assistant medical officers, and we are glad to note that one of these is a lady. The salaries received by the medical staff are not by any means large. Certainly the Asylum is a small one; but we doubt whether the salaries given are sufficient to retain the services of the best medical officers, and we hope the next report will show an increase in the three salaries. The weekly charge to the Union is 9s. The Committee say they "desire to express their sense of the care and attention which have been bestowed on the management of this large institution by Dr. Morrison and the medical staff."

